# Comment on “Discussion on the Timing of Balloon Occlusion of the Abdominal Aorta during a Caesarean Section in Patients with Pernicious Placenta Previa Complicated with Placenta Accreta”

**DOI:** 10.1155/2018/9493878

**Published:** 2018-07-19

**Authors:** Shigeki Matsubara

**Affiliations:** Department of Obstetrics and Gynecology, Jichi Medical University, Tochigi, Japan

The drier the surgical field, the better: this holds true for every surgery. The treatment of abnormally invasive placenta (AIP: accreta, increta, percreta) is still challenging, and aortic balloon occlusion (ABO) has attracted obstetricians' attention: it may reduce bleeding during surgery. Zhu et al. [1] demonstrated interesting data. In presurgically suspected AIP patients with the placenta covering the previous cesarean scar, perinatal outcomes were compared between two strategies: ABO performed “before” versus “after” infant delivery. Bleeding during surgery was significantly reduced “before” compared with “after” (413 versus 810 mL, respectively) with no difference in the neonatal outcomes. Based on these data, Zhu et al. concluded that ABO “before” uterine incision is better than “after” it. I have some clarifications and concerns.

Zhu et al.'s strategy was an “extirpative approach” with the aid of ABO. Treatment strategies for AIP are divided into four [2]: (i) forcible placental removal (extirpative approach), (ii) partial uterine resection with the placenta attached to it, (iii) placenta left* in situ* approach, and (iv) cesarean hysterectomy. The gold standard of AIP treatment is cesarean hysterectomy [2, 3]; however, this precludes fertility, and, thus, “uterus-preserving strategies (i, ii, iii)” may be used in some selected cases [2]. Of note, an “extirpative approach”, which used to be widely employed, has now been fundamentally abandoned and rather prohibited in the treatment of presurgically suspected AIP since it frequently causes marked bleeding and necessitates hysterectomy [2]. However, interventional radiology created “room” to perform an “extirpative” approach. ABO may have shed new light on the once-abandoned extirpative approach, which Zhu et al. demonstrated: irrespective of before versus after, extirpative approach did not cause marked bleeding and preserved uterus in almost all patients. This is important before the discussion of before versus after.

My first concern regards the study design. One specialist (Doctor A) performed “before” and the other specialist (Doctor B) performed “after”. Usually, medical study design must be as follows: “the same team/doctor(s)” performs both approaches, and, then, comparison is made between the two. Consider this scenario: Doctor A, irrespective of “before” versus “after”, usually performs surgery with less bleeding than Doctor B. The surgical outcome, especially the amount of bleeding, is markedly influenced by each surgeon's individual experience/skill [4]. Zhu et al. stated, “The two doctors have different surgical experiences”. At least the following should be demonstrated: both Doctor A and Doctor B usually perform surgery (surgery other than this specific surgery) with approximately the same amount of bleeding. The present study design arouses a simple question whether comparison has been made between “before”/“after” or Doctor A/B.

The second concern regards the duration of balloon occlusion. Firstly, this duration was not described. When we use ABO, we empirically set its occlusion limit as 20 minutes. Undoubtedly, “before” requires much longer occlusion than “after”. The “admissible” time/duration for ABO occlusion has yet to be determined but it may exist in a spectrum and may not be a yes/no matter. The shorter the occlusion duration, the better. Secondly, in the “after” group, Zhu et al. performed the procedure in the following order: (i) uterine incision (avoiding the placenta) → (ii) infant delivery → (iii) balloon occlusion → (iv) closing the uterine incision. In my four-decade experience, at the infant-delivery stage, the amount of bleeding is not large or at least controllable by simply clamping the incision edge by forceps, since the placenta has yet to be separated or destroyed. The balloon occlusion should be performed after (iv) closing the uterine incision, which may delay the timing of occlusion by several minutes. Zhu et al. stated that “no blood” environment can reduce bleeding during surgery. This is true; however, I wonder whether 413 mL (before) versus 810 mL (after) makes any practical difference, when we take into account the fact that “before” necessitates a longer ischemic duration/time, and so may increase the possibility of ischemic adverse events.

The third concern is that one maternal death occurred in the “before” group. “A massive retroperitoneal hematoma formed by the abdominal aorta dissection” caused the maternal death. The situation surrounding this aorta dissection was not described in detail. I wonder whether this may have been associated with the longer occlusion employed in the “before” group.

I commend Zhu et al. for their pioneering efforts. However, “no blood” may be very difficult or even impossible, considering this disease character. We must weigh the balance between the amount of bleeding and possible adverse events caused by a “longer” occlusion time. In my opinion, the timing of balloon occlusion should be decided in a patient-by-patient manner [5]. It should be inflated just prior to predicted marked bleeding. The drier the surgical field, the better; however, how dry it should be is another matter.

## Abbreviations

ABO: Aortic balloon occlusion
AIP: Abnormally invasive placenta.
